# Integrated system for detection and molecular characterization of circulating tumor cells

**DOI:** 10.1371/journal.pone.0237506

**Published:** 2020-08-13

**Authors:** Yusuke Takahashi, Kentaro Shirai, Yuichi Ijiri, Eri Morita, Tomokazu Yoshida, Shigeki Iwanaga, Masatoshi Yanagida

**Affiliations:** Department of Central Research Laboratories, Sysmex Corporation, Takatsukadai, Nishi-ku, Kobe, Japan; Texas Tech University Health Science, Lubbock, UNITED STATES

## Abstract

Circulating tumor cells (CTCs) invade blood vessels in solid tumors and promote metastases by circulating in the blood. CTCs are thus recognized as targets for liquid biopsy and can provide useful information for design of treatments. This diagnostic approach must consider not only the number of CTCs but also their molecular and genetic characteristics. For this purpose, use of devices that enrich CTCs independent of these characteristics and detectors that recognize various CTC characteristics is essential. In the present study, we developed a CTC detection system comprising ClearCell FX and ImageStream Mark II. We clarified the analytical performance of this system by evaluating recovery rate, lower limits of detection, and linearity. These parameters are critical for detecting rare cells, such as CTCs. We tested these parameters using three cell lines with different expression levels of the epithelial marker-epithelial cell adhesion molecule (EpCAM) and spiked these cells into whole-blood samples from healthy donors. The average recovery rate and lower limit of detection were approximately 40% and five cells/7.5 mL of whole blood, respectively. High linearity was observed for all evaluated samples. We also evaluated the ability of the system to distinguish between normal and abnormal cells based on protein expression levels and gene amplification and found that the system can identify abnormal cells using these characteristics. The CTC detection system thus displays the ability to distinguish specific characteristics of CTC, thereby providing valuable information for cancer treatment.

## Introduction

Circulating tumor cells (CTCs) are solid cancer-derived cells that circulate in blood [1–3]. CTCs are essential for cancer metastasis [4,5]. The relationship between CTCs and cancer metastasis has been studied for various types of cancer, and the presence of CTCs is a substantial risk factor for cancer metastasis and reduced survival [6–10]. Furthermore, CTCs have attracted attention as targets for liquid biopsy because they display characteristics that reflect primary and metastatic lesions [11]. Thus, CTC testing should be useful for drug selection and monitoring therapeutic efficacy [12–14]. Cancer diagnosis and characterization using CTCs require an evaluation of both numbers of cells and their molecular and genetic abnormalities.

CTC detection in blood samples is difficult because of the rarity of CTCs. The abundance ratio of CTCs to white blood cells is approximately 10^2^ per 10^7^ leukocytes [6,15]. Reliable results require removal of normal blood cells to increase CTC purity. CTC detection is also difficult because of cancer heterogeneity [11,16]. Cancer cells often undergo epithelial–mesenchymal transition, during which their properties change [17–19]. Therefore, CTC enrichment using epithelial cell markers, such as EpCAM, cannot recover CTC with mesenchymal characteristics [20–23]. Obtaining accurate counts and characteristics cannot depend on molecular expression for enrichment.

The device used for CTC detection is also important. A microscope or flow cytometer is typically selected for this task [24–27]. A fluorescence microscope can identify CTCs based on images, thereby showing high specificity. However, images must be acquired at a low magnification to maintain high throughput. Thus, fluorescence microscopy is not suitable for techniques that require high resolution, such as molecular localization analysis or fluorescence *in situ* hybridization (FISH). Flow cytometers are high-throughput detection devices but do not generate cell images showing molecular localization and cell morphology. These limitations reduce detection accuracy. Understanding the characteristics of CTCs requires detectors with high specificity and throughput.

We developed such a CTC detection system by combining a CTC enrichment device that does not rely on molecular expression with a CTC detection device that yields high-throughput and high-resolution images based on flow cytometry. For CTC enrichment, we adopted ClearCell FX (Biolidics, Mapex, Singapore), a spiral microfluidic device that separates CTCs from whole blood based on cell size [28,29]. This size-based enrichment method does not rely on the molecular characteristics of CTCs and can recover almost all CTCs rapidly and efficiently. ImageStream Mark II (imaging flow cytometer) (Luminex, Northbrook, USA) was selected for CTC detection. This imaging flow cytometer is a detector that combines the high throughput of flow cytometry measurements with high-resolution imaging [30,31]. Magnification of objective lenses can be set up to 60 times, allowing cells to be distinguished according to localization of molecules or number and localization of bright spots visualized on FISH [32]. Furthermore, the instrument is capable of multicolor fluorescence detection of up to 12 colors and is a measurement platform suitable for evaluating molecular or genetic characteristics.

To date, no reports are available that evaluate CTC detection using a combination of ClearCell FX and an imaging flow cytometer. Therefore, we aimed to clinically test CTC detection by combining the advantages of these two devices. Furthermore, we assessed recovery rate, lower limits of detection, and linearity of the system using cancer cell lines with or without epithelial phenotypic characteristics. Finally, we considered immunostaining and FISH as tools for use with the system.

## Materials and methods

### Experimental design

This study was conducted to show the analytical performance of a CTC detection system. Analytical performance refers to recovery rate, linearity, lower limits of detection, and discrimination between white blood cells (WBCs) and CTCs. Three types of cell lines with different expression levels of EpCAM were prepared and spiked into whole blood from healthy donors to mimic the blood of cancer patients. Blood samples were processed with an enrichment device, ClearCell FX, to increase concentrations of CTCs. After enrichment, samples were stained by FISH or fluorescent-labeled antibodies. Stained samples were processed with an imaging flow cytometer to assess an analytical performance. Finally, we evaluated opportunities to further improve system performance.

### Cell lines

Breast cancer cell lines SK-BR-3 and HCC1569 and non-small-cell lung cancer cell line A549 were obtained from the American Type Culture Collection (Manassas, USA). SK-BR-3 was cultured in Macoy’s 5A medium (SIGMA-Aldrich, Tokyo, Japan) with 10% fetal bovine serum (SIGMA) and 1% Antibiotic-Antimycotic (Thermo Fisher SCIENTIFIC, Waltham, USA). HCC1569 and A549 were cultured in Dulbecco’s Modified Eagle Medium (SIGMA) with the same composition as complete medium for SK-BR-3. Passaging was performed twice a week with a four-fold dilution.

### EpCAM-positive and -negative cell lines

For preparing EpCAM-negative or weakly positive cell lines, A549 and HCC1569 cells were cultured with complete medium with 5 ng/mL of transforming growth factor-β (TGF-β) (PeproTech, Rocky Hill, USA) for 1 month. Passaging was performed twice a week with a four-fold dilution. TGF-β-treated HCC1569 cells were applied to an EpCAM-Magnetic Beads column (Clone HEA-125; Militenyi Biotec, Tokyo, Japan), and the flow-through fraction was collected. This process was followed the manufacturer’s recommended protocol. TGF-β-treated A549 cells are abbreviated “A549TT” and EpCAM-depleted HCC1569 cells, “HCC1569DP.” SK-BR-3 was selected as an EpCAM-positive cell line. EpCAM expression in each cell line was determined by staining cells with Phycoerythrin (PE)-labeled anti-EpCAM antibody (Clone HEA-125; Militenyi Biotec, Tokyo, Japan) and measuring fluorescence with the imaging flow cytometer. EpCAM expression level was analyzed using the Mann–Whitney U-test. For spiking experiments, all three cell lines were harvested using 0.25% trypsin/ethylenediaminetetraacetic acid (Thermo Fisher SCIENTIFIC), suspended in CELLBANKER1 (ZENOAQ, Fukushima, Japan), and stored at −80°C. Stored cell lines were thawed before spiking experiments.

### Blood collection

The study was performed with the approval of the Sysmex Research Ethics Examination Committee. Institutional Review Board approval was obtained on May 25, 2017. The approval number was 2017–10. Consent to participate in the study was obtained in writing. Whole-blood samples were obtained from 66 healthy donors (Seishin-Center, Hyogo, Japan) between May and November 2017. All participants were enrolled using Institutional Review Board-approved protocols and provided informed consent. Whole-blood samples (10 mL) were collected into two ethylenediaminetetraacetic acid blood collection tubes (TERUMO, Tokyo, Japan). After collection, samples were used for the spiking experiments within 1 day.

### CellHunt dye labeling

Cryopreserved cells were incubated for 5 min at 37°C and suspended in fetal bovine serum-free medium. After centrifugation at 500 × g for 3 min, the supernatant was discarded and stained cells with fetal bovine serum-free medium containing 1 mM CellHunt dye (Setareh Biotech, Eugene, USA) for 90 min. During staining, the sample was kept at 37°C and 5% CO_2_. CellHunt dye was used to measure the number of spiked cells and to verify that cells detected by imaging cytometry were spiked cells.

### Spiking experiment: Separation and concentration of CTC

CellHunt dye-stained cells were placed in 96-well plates (AGC TECHNO GLASS, Shizuoka, Japan). After 15 min, numbers of cells were counted under a fluorescence microscope, IX83 (Olympus, Tokyo, Japan). This cell count was used as a reference number for spiked cells. For spiking, cells were resuspended with 100 μL of PBS and blood samples were prepared by adding 0, 5, 15, 30, and 150 cells to 7.5 mL of whole blood. Samples were placed into the ClearCell FX system, and spiked cells were enriched following the recommended protocol.

### Spiking experiment: *HER2* FISH

After enrichment, recovered samples containing SK-BR-3 and HCC1569DP cells were treated with a *HER2* FISH probe (Oxford Gene Technology, Begbroke, UK). The probe consists of a probe for the *HER2* gene and a probe for the centromere 17, and probes are labeled with fluorescein isothiocyanate and Texas Red, respectively. Carnoy’s solution was prepared for cell fixation and permeabilization as described previously [32]. Cells were resuspended in 30% (v/v) Carnoy’s solution and incubated at 4°C for 30 min. Cells were then resuspended in 70% (v/v) Carnoy’s solution and incubated at 4°C for 30 min. Cells were centrifuged at 200 × g for 10 min, and the supernatant was discarded. Cells were washed twice with 1 mL of phosphate-buffered saline (Wako, Osaka, Japan) to discarded Carnoy’s solution. Cells were then hybridized with the Cytocell *HER2* FISH probe (Oxford Gene Technology). After hybridization, washing was performed following the recommended protocol from Oxford Gene Technology. Cells were centrifuged at 500 × g for 1 min, and the supernatant was discarded. Finally, cells were washed and treated with 100 nM DRAQ5 for 10 min for nuclear staining.

### Spiking experiment: Immunostaining for cytokeratin and CD45

After enrichment for A549TT cells, recovered samples were incubated in phosphate-buffered saline (FUJIFILM Wako, Osaka, Japan) containing 1% bovine serum albumin (Lampire, Pipersville, USA) for 20 min and centrifuged at 500 × g for 10 min. Samples were then incubated with 100 μL of antibody solution (Alexa Fluor 647-labeled anti-cytokeratin antibody [Clone C11; Cell Signaling Technologies, Danvers, USA]) (1:20) and PE-Cyanin 7-labeled anti-CD45 antibody (Clone HI-30; BioLegend, San Diego, USA) (1:50) containing 0.1% HOECHST33342 and 1% bovine serum albumin in phosphate-buffered saline for 60 min at room temperature. Cytokeratin was used as a marker for spiked cells, and CD45 was used as a marker of WBCs.

### Spiking experiment: CTC detection

Imaging flow cytometer conditions for measuring *HER2* FlowFISH samples were as follows: magnification of the objective lens, 40×; flow rate, middle; and extended depth filter, on. Laser power for wavelength 405 nm was set to 120 mW; for 488 nm, 200 mW; and for 592 nm, 300 mW. Conditions for measurement of immunostained samples with the imaging flow cytometer were as follows: laser power at 405 nm was set to 40 mW; for 488 nm, 4 mW; for 561 nm, 200 mW; and for 642 nm, 150 mW.

### Spiking experiment: Statistical analysis

The analysis software, IDEAS, was used to evaluate recovery rate, limits of detection, linearity, and CTC/WBC discrimination performance of the CTC detection system. CellHunt dye-positive cells were used as a baseline reference for spiked cells, and CellHunt dye-negative cells were used as a reference for normal cells. Recovery rate was calculated as the number of CellHunt dye-positive cells divided by that of spiked cells. Samples with 150 cells spiked were used for analysis to achieve high accuracy. Lower limits of detection were calculated as the minimum spiking condition for which the value obtained by subtracting two standard deviations from the average number of CellHunt dye-positive cells was ≥1. Linearity was evaluated using coefficients of determination in first-order linear regression equations from scatter plots of numbers of detected cells against numbers of spiked cells. Discrimination performance between CTCs and leukocytes used sensitivity calculated by dividing the number of *HER2* or cytokeratin-positive cells by the number of CellHunt dye-positive cells. Specificity was calculated by dividing numbers of *HER2* or cytokeratin-negative cells by the number of CellHunt dye-negative cells.

### Recovery rates

Evaluation of the recovery rate used a whole-blood sample spiked with 150 CellHunt dye-stained SK-BR-3 cells. The recovery rate of after enrichment was calculated as the number of CellHunt dye-positive cells in ClearCell FX recovery samples divided by the number of spiked cells. Recovery rate of FISH was calculated as the number of CellHunt dye-positive cells after FISH divided by that of CellHunt dye-positive cells after enrichment. Recovery rate of imaging flow cytometry was calculated as the number of CellHunt dye-positive cells obtained divided by the number of CellHunt dye-positive cells after FISH. Numbers of CellHunt dye-positive cells after enrichment and FISH were counted under a fluorescence microscope, IX83.

## Results

### CTC detection system concept

**Fig 1:**
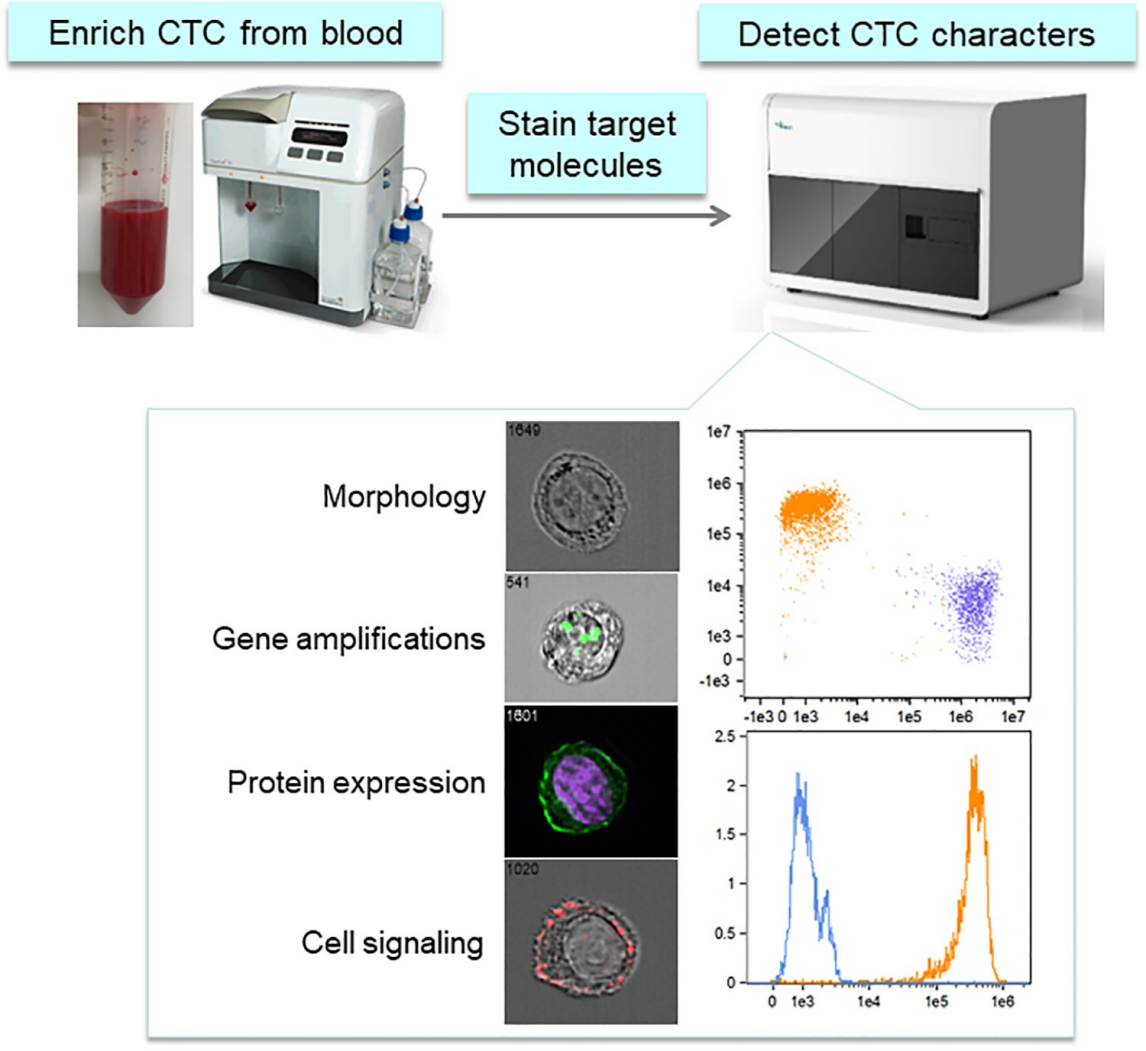
Image of the CTC detection system. ClearCell FX is used for CTC enrichment, and ImageStream Mark II (imaging flow cytometer) is used for CTC characterization, e.g., cell morphology, gene abnormality, protein expression, and intracellular signaling. https://doi.org/10.6084/m9.figshare.12432530.v2.

CTCs have attracted attention as targets for liquid biopsy because they display characteristics of primary and metastatic lesions [11]. However, CTC detection in blood samples is difficult because of rarity and heterogeneity [15,16]. The CTC detection system is based on improving cancer treatments by stably recovering heterogeneous CTCs and detecting molecular and genetic characteristics with high specificity. The CTC detection system comprises a device that can enrich CTCs regardless of protein expression and a detection device that can determine morphology, molecular characteristics, and genetic abnormality of CTCs with high throughput and specificity (Fig 1). For system, the ClearCell FX system was selected for CTC enrichment, and an imaging flow cytometer was adopted for CTC characterization. The system can complete CTC measurements from a single sample within 6 h.

### Sample preparation for spiking experiments

**Fig 2:**
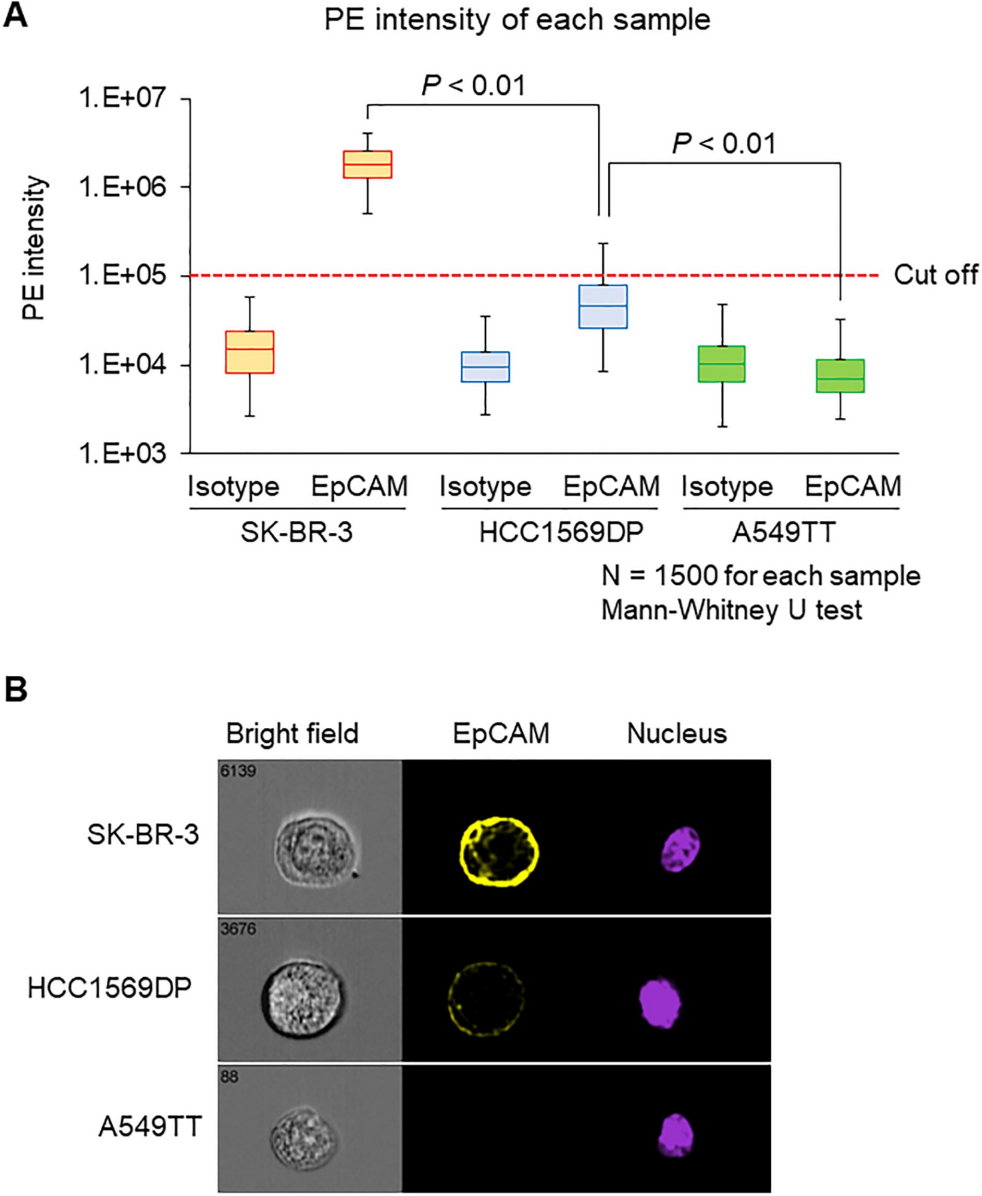
Evaluation of EpCAM expression level. Each cell line was stained with a PE-anti-EpCAM antibody or an isotype control antibody. Each sample contained 1500 measured cells. (A) Intensity of PE fluorescent signals. Data are presented as a box-whisker plot. The lower whisker indicates the 2.5^th^ percentile, and the upper whisker indicates the 99.5^th^ percentile. The fluorescence signal cutoff of PE was set so that the positive rate of isotype control in all three cell lines was 0%. Data were analyzed using the Mann–Whitney U-test. (B) Immunostaining images obtained by imaging flow cytometry. https://doi.org/10.6084/m9.figshare.12433157.v2.

A CTC detection system can conceptually enrich CTCs regardless of their protein expression and characterize CTCs using an imaging flow cytometer. EpCAM expression is highly variable within CTC populations [33]. Three types of cancer cell lines were selected to demonstrate the usefulness of our system: SK-BR-3 as high-, HCC1569DP as medium-, and A549TT as no-EpCAM-expressing cell lines. Imaging flow cytometry was used to confirm that EpCAM expression in each cell line was performed. The percentage of EpCAM-positive cells was 100% for SK-BR-3, 18% for HCC1569DP, and 0% for A549TT (Fig 2A). Median fluorescence signal intensity of EpCAM for SK-BR-3, HCC1569DP, and A549TT was 1805193, 46565, and 4944, respectively (Fig 2A). A representative photograph of each cell line imaged using imaging flow cytometry showed that EpCAM is localized at the cell membrane (Fig 2B). The cell lines used for spiking experiments thus display different EpCAM expression levels.

### Recovery rate, lower limits of detection, and linearity of the CTC detection system

**Fig 3:**
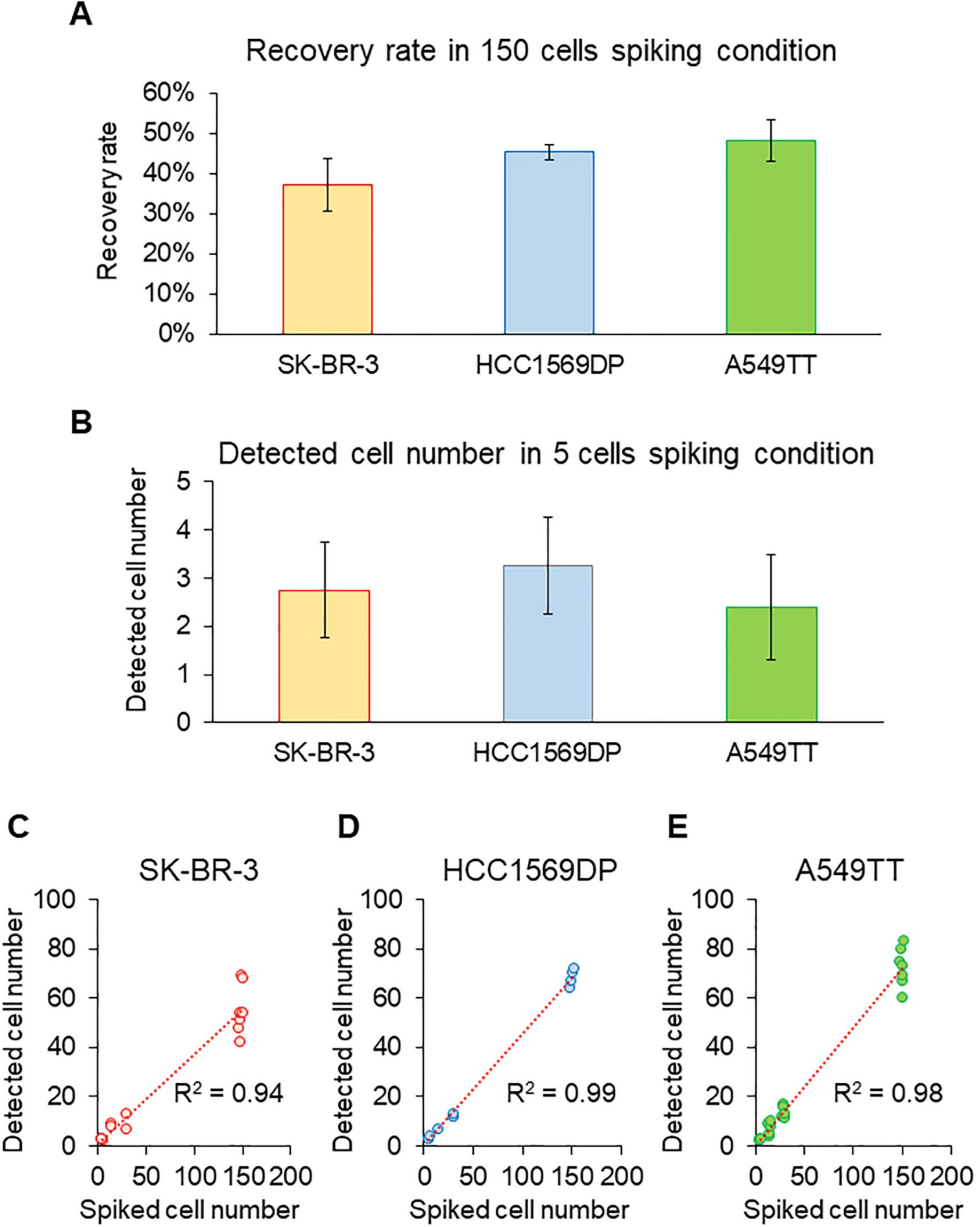
Recovery rate, lower limit of detection, and linearity. (A) Recovery rates of SK-BR-3 (n = 7), HCC1569DP (n = 4), and A549TT (n = 7). Recovery rate was calculated for 150 cells spiked into whole blood. Data are presented as mean ± 1 standard deviation (SD). (B) Lower limits of detection of SK-BR-3 (n = 4), HCC1569DP (n = 4), and A549TT (n = 5). Lower limits of detection were calculated as the minimum spiking condition where the value obtained by subtracting 2 SD from the average number of detected cells is ≥1. Data are presented as mean ± 2 SD. (C–E) Linearity of SK-BR-3 (n = 21), HCC1569DP (n = 19), and A549TT (n = 26). Linearity was evaluated by the coefficient of determination in the first-order linear regression equation of a scatter diagram that plots numbers of detected cells against numbers of spiked cells. https://doi.org/10.6084/m9.figshare.12433265.v3.

Analytical performance was evaluated using a spiking experiment. Average recovery rate of SK-BR-3 cells was 37% (CV 18%), that of HCC1569DP cells was 45% (CV 5%), and that of A549TT cells was 48% (CV 11%) (Fig 3A). The lower limit of detection was five cells/7.5 mL of whole blood across all samples (Fig 3B). Coefficients of determination were 0.98 (n = 21) for SK-BR-3, 0.96 (n = 19) for HCC1569DP, and 0.98 (n = 26) for A549TT. Thus, results for all cell lines showed high linearity (Fig 3C–3E). These results indicate that the CTC detection system exhibits a stable recovery rate, high detection sensitivity, and high linearity without regard to EpCAM expression.

### Detection performance of cytokeratin immunostaining or *HER2* FISH staining

**Fig 4:**
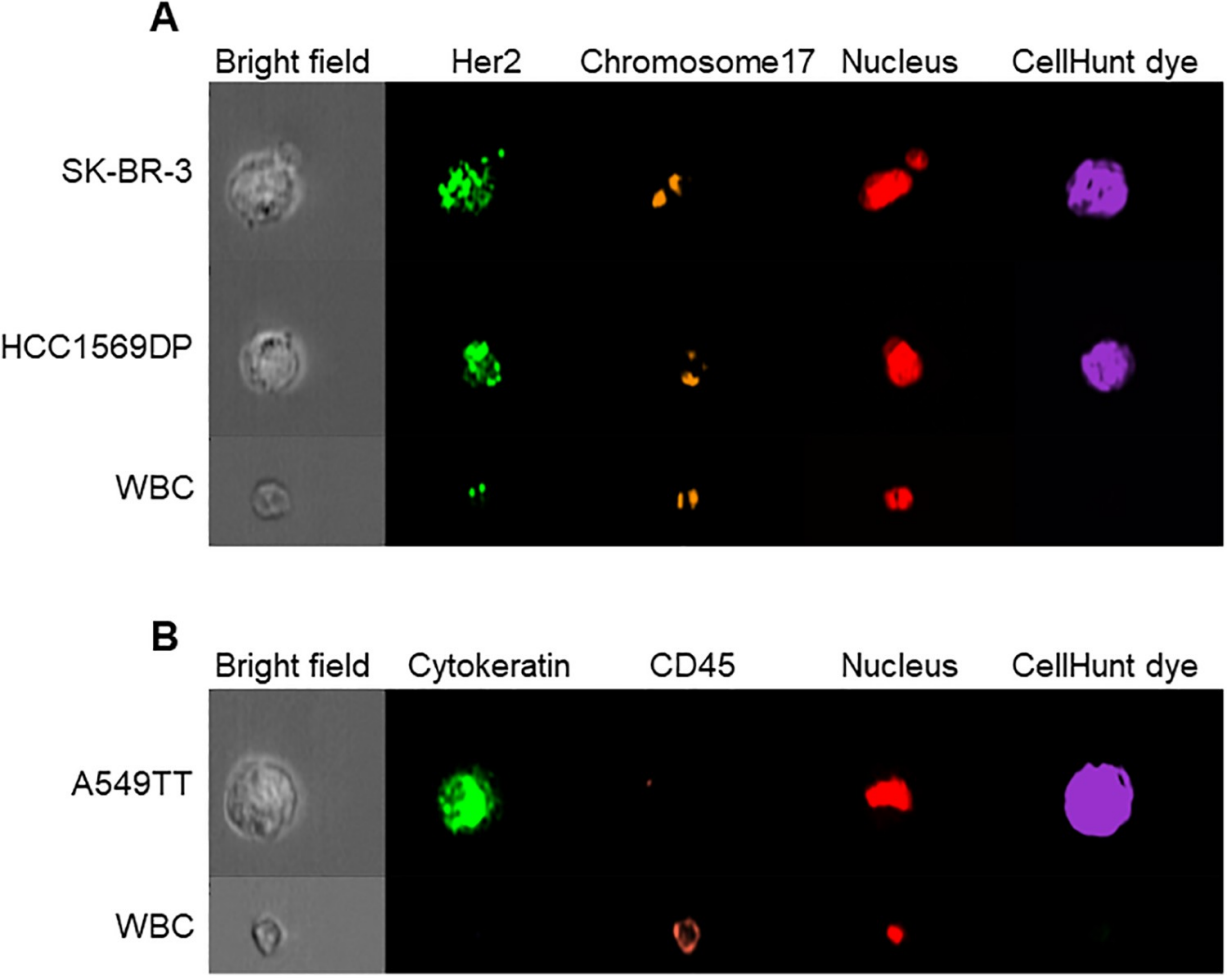
Cell images of each cell line and white blood cells obtained by imaging flow cytometry. (A) FISH staining image of SK-BR-3, HCC1569, and white blood cells. *HER2* (green) and chromosome 17 (red) were stained using the Cytocell *HER2* FISH probe, and the nucleus was stained using DRAQ5. (B) Immunostaining image of A549TT and white blood cells. Cytokeratin (green) and CD45 (red) were stained by specific antibodies. The nucleus was stained with Hoechst 33342. https://doi.org/10.6084/m9.figshare.12433484.v1.

The sensitivity and specificity for the identification of cytokeratin expression and *HER2* amplification were evaluated to determine performance of the CTC detection system for measuring protein expression and gene abnormality. After FISH staining, *HER2* gene spots amplified against WBCs were observed for SK-BR-3 and HCC1569DP cells (Fig 4). After immunostaining, cytokeratin fluorescence was observed only for A549TT cells (Fig 4). Sensitivity and specificity for anti-cytokeratin immunostaining were 86% and 100%, respectively (Table 1). Sensitivity and specificity for SK-BR-3 cells were 87% and 100% for *HER2* FISH, respectively (Table 1). Analogous results for HCC1569DP cells were 69% and 100%, respectively (Table 1). The CTC detection system thus displays detection performance and can measure CTC characteristics such as protein expression and gene abnormality.

**Table 1:** Sensitivity and specificity of the CTC detection system.

Spiked cell line	Detection	Sensitivity	Specificity
**SK-BR-3**	FISH for *HER2*	87%	100%
**HCC1569DP**	FISH for *HER2*	69%	100%
**A549TT**	Immunostaining for cytokeratin	86%	100%

### Evaluation of recovery rates

**Fig 5:**
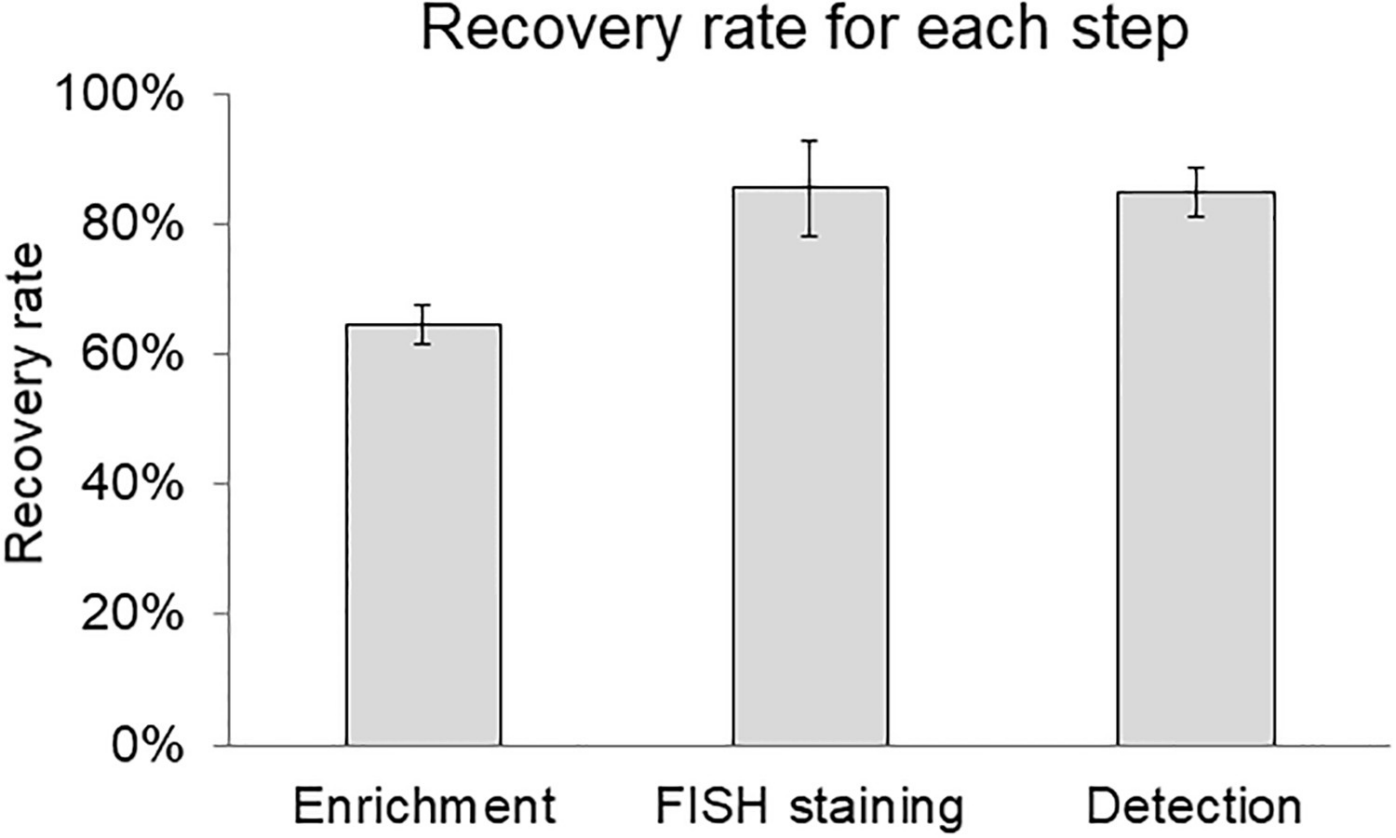
Recovery rates in each step of the CTC detection system. CellHunt dye-stained SK-BR-3 (150 cells) were spiked into 7.5 mL of whole blood and evaluated for recovery rate in each step of the process (n = 3). Data are presented as mean ± 1 SD. https://doi.org/10.6084/m9.figshare.12433493.v1.

High recovery rates are required to detect CTCs and were assessed at enrichment, staining, and detection steps. The average recovery rate for CTC enrichment was 64.6% (CV 4.7%); for staining, 85.5% (CV 8.6%); and for detection, 84.8% (CV 4.5%) (Fig 5). Recovery of CTC was lowest in the initial enrichment step. CTC enrichment step requires improvement for recovery of CTC.

## Discussion

Generally, CTC detection methods using antibodies have a recovery rate of ≥80% [6,34], which is considerably higher than that of the CTC detection system. However, CTC detection using antibodies cannot detect epithelial marker-negative CTCs such as A549TT and HCC1569DP cells. Furthermore, lower limits of detection for our system were similar to CTC detection methods using antibodies.

The spiking experiments showed that SK-BR-3 cells display a lower recovery rate than the other two cell lines, a factor likely related to the smaller size of SK-BR-3 cells. The mean size ± 1 SD of SK-BR-3 before spiking into blood was 17.2 ± 4.8 μm, whereas the size of HCC1569DP cells was 19.6 ± 3.0 μm (S1 Fig). In the case of CTCs with a smaller size than SK-BR-3, recovery rate is likely to be lower. ClearCell FX enriches cells, in part, based on size. This problem can overcome with a novel spiral chip, which has smaller size cutoff than existing chip. Smaller size cutoff increases WBC contamination in enriched samples, however, cytometer allows up to 10 times amount the WBC contamination because of its effect of high-throughput.

The lower sensitivity of HCC1569DP cells than that of SK-BR-3 cells is due to *HER2* copy number variation of (Table 1) [35]. For detecting CTCs with low *HER2* amplification, a probe with sufficient luminance is needed along with a protocol to achieve acceptable staining efficiency.

Cells judged to be cytokeratin-negative despite being positive for CellHunt dye were detected (Table 1). We found that hemolysis reduced the efficiency of immunostaining (S2 Fig). Hemolysis is one factor that can cause false negative results in CTC detection. Thus, development of the CTC enrichment method that does not require hemolysis will improve CTC detection.

Our results further show that enrichment is the limiting factor for improving recovery in the entire CTC detection system. Decreasing the size cutoff of the CTC enrichment chip is likely to increase WBC contamination, and the performance of the imaging flow cytometer will likely tolerate this increase in WBC contamination. Temperature and buffer type can also be factors affecting recovery rate. These parameters also need to be further tested.

The present study shows clearly that the CTC detection system demonstrates high analytical performance. Clinical research is needed to demonstrate the clinical value of this system and further improve system recovery rate.

## Supporting information

S1 Fig Cell size distribution of SK-BR-3 (A) and HCC1569DP (B). Cell lines were measured with an imaging flow cytometer. Cell diameter was calculated from the formula of the area of the circle assuming that the cell was perfectly round. Cell size distribution is shown as mean ± 1 SD. https://doi.org/10.6084/m9.figshare.12433499.v1.
(TIF)

S2 Fig Evaluation of the effect of hemolytic treatment on immunostaining. (A) Non-hemolysis-treated A549TT stained with isotype control antibody. (B) Hemolysis-treated A549TT stained with isotype control antibody. (C) Non-hemolysis-treated A549TT stained with Alexa Fluor 647-labeled anti-cytokeratin antibody. (D) Hemolysis-treated A549TT stained with Alexa Fluor 647-labeled anti-cytokeratin antibody. https://doi.org/10.6084/m9.figshare.12433508.v1.
(TIF)
