# Supplementary figures and images for: Integrated system for detection and molecular characterization of circulating tumor cells
Source: PLoS One. 2020 Aug 13;15(8):e0237506. doi: 10.1371/journal.pone.0237506 (PMC7425940; doi:10.1371/journal.pone.0237506)

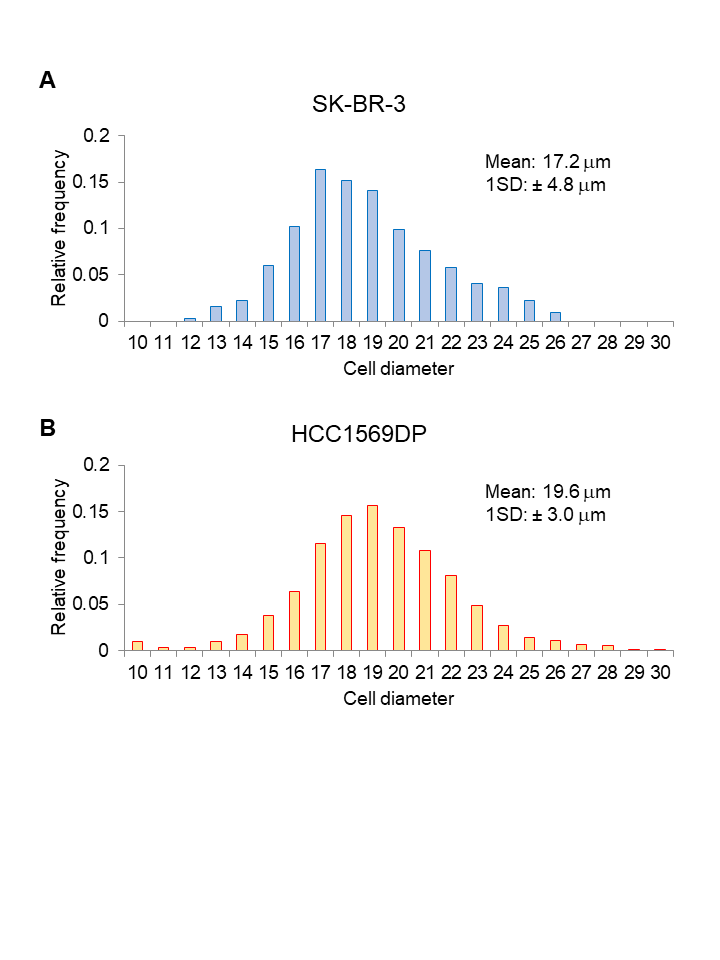

Supplement: S1 Fig — Cell lines were measured with an imaging flow cytometer. Cell diameter was calculated from the formula of the area of the circle assuming that the cell was perfectly round. Cell size distribution is shown as mean ± 1 SD. https://doi.org/10.6084/m9.figshare.12433499.v1. (TIF) [file pone.0237506.s001.tif]

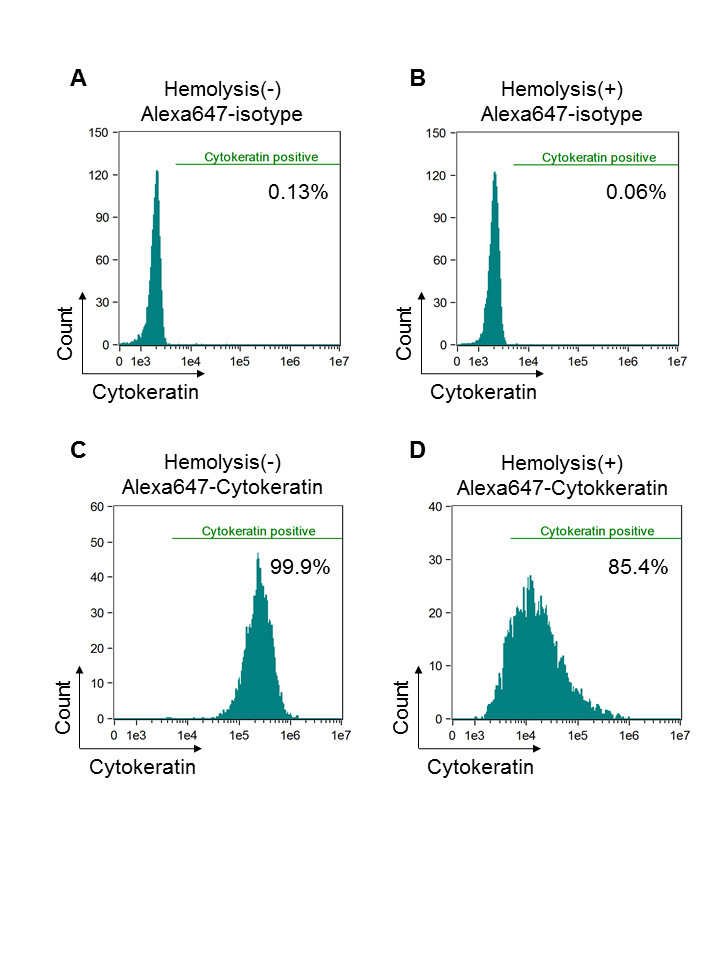

Supplement: S2 Fig — (A) Non-hemolysis-treated A549TT stained with isotype control antibody. (B) Hemolysis-treated A549TT stained with isotype control antibody. (C) Non-hemolysis-treated A549TT stained with Alexa Fluor 647-labeled anti-cytokeratin antibody. (D) Hemolysis-treated A549TT stained with Alexa Fluor 647-labeled anti-cytokeratin antibody. https://doi.org/10.6084/m9.figshare.12433508.v1. (TIF) [file pone.0237506.s002.tif]
